# MiR103a-3p and miR107 are related to adaptive coping in a cluster of fibromyalgia patients

**DOI:** 10.1371/journal.pone.0239286

**Published:** 2020-09-17

**Authors:** Alexandra Braun, Dimitar Evdokimov, Johanna Frank, Claudia Sommer, Nurcan Üçeyler

**Affiliations:** Department of Neurology, University of Würzburg, Würzburg, Germany; Kunming University of Science and Technology, CHINA

## Abstract

**Background:**

MicroRNA (miRNA) mainly inhibit post-transcriptional gene expression of specific targets and may modulate disease severity.

**Objective:**

We aimed to identify miRNA signatures distinguishing patient clusters with fibromyalgia syndrome (FMS).

**Subjects and methods:**

We previously determined four FMS patient clusters labelled “maladaptive”, “adaptive”, “vulnerable”, and “resilient”. Here, we cluster-wise assessed relative gene expression of miR103a-3p, miR107, miR130a-3p, and miR125a-5p in white blood cell (WBC) RNA of 31 FMS patients and 16 healthy controls. Sum scores of pain-, stress-, and resilience-related questionnaires were correlated with miRNA relative gene expression. A cluster-specific speculative model of a miRNA-mediated regulatory cycle was proposed, and its potential targets verified by the online tool “*target scan human”*.

**Results:**

One-way ANOVA revealed lower gene expression of miR103a-3p, miR107, and miR130a-3p in FMS patients compared to controls (p < 0.05). Follow-up post-hoc tests indicated the highest peak of gene expression of miR103a-3p for the adaptive cluster (p < 0.05), i.e. in patients with low disability in all symptom categories. Gene expression of miR103a-3p correlated with FMS related disability and miR107 with the score “physical abuse” of the trauma questionnaire (p < 0.05). Target scan identified sucrose non-fermentable serine/threonine protein kinase, nuclear factor kappa-b, cyclin dependent kinase, and toll-like receptor 4 as genetic targets of the miR103a/107 miRNA family.

**Conclusion:**

We show an association between upregulated gene expression of miR103a, tendentially of miR107, and adaptive coping in FMS patients. Validation of this pair of miRNA may enable to identify a somatic resilience factor in FMS.

## Introduction

Fibromyalgia syndrome (FMS) is characterized by chronic widespread pain and additional symptoms such as depressive mood, sleep disorders, and gastrointestinal problems [1]. The pathophysiology of FMS is incompletely understood, but there is evidence that higher expression of pro-inflammatory cytokines and experience of traumatic events during childhood may be associated with the development and severity of FMS [2, 3].

MicroRNA (miRNA) are small noncoding RNA that mostly function in post-transcriptional regulation by translational inhibition, silencing and mRNA destabilization of specific target genes [4]. MiRNA are involved in the pathophysiology of inflammation, pain, and mood disorders [5] and may characterize patient subgroups in heterogeneous disorders [6].

In FMS patients, the expression of miRNA has been investigated in body fluids [7–10]. For instance, miR107 and miR103a-3p expression was reduced in blood samples of FMS patients compared to healthy controls, and miR103a-3p correlated with pain and sleep duration [11]. Given the vast phenotypic heterogeneity of FMS, we previously conducted a factor analysis and labelled clusters as “maladaptive”, “adaptive”, “vulnerable”, and “resilient” [12]. The main characteristics of these clusters were “dysfunctional coping and pro-inflammatory cytokine pattern”, “active coping and low disability”, “high catastrophizing and high negative affect”, and “resilient coping (reappraisal) with high traumatic stress”. We now asked whether miRNA signatures might distinguish these clusters.

Studies in patients suffering from inflammatory (e.g. preeclampsia) and (post-traumatic) stress-related syndromes (e.g. post-traumatic stress disorder) identified some miRNA and their genetic targets, e.g. the miR103a/107 miRNA family as involved in different pathways, e.g. the STAT6/IL4 anti-inflammatory pathway [13] or the SNRK / NF-κB / p65 signaling pathway [14]. MiR107 is linked to childhood trauma [15] and has a regulatory role in inflammation [16, 17] by targeting cycline dependent kinases (CDK) and toll like receptors (TLR) [18, 19]. Activated TLR4 was shown to induce secretion of tumor necrosis factor-alpha (TNF) in patients with renal sepsis [20] and to attenuate adaptive thermogenesis in obese mice via endoplasmatic reticulum stress. Pro-inflammatory processes are known to be a driver of chronic pain [21] and have an impact on mental issues [22].

Based on these findings, we hypothesized that an upregulation of miR103a-3p, miR107, miR130a-3p, and miR125a-5p might promote adaptive coping in the adaptive and resilient cluster of our FMS cohort. We report on higher gene expression of miR103a-3p and miR107 in the adaptive cluster of FMS patients compared to the maladaptive, vulnerable, and resilient cluster. Our data may facilitate the discovery of somatic factors of resilience and adaptation in FMS patients in further studies.

## Materials and methods

### Subjects and ethics

FMS patients and healthy controls were recruited between 2014 and 2018 at the Department of Neurology of the University Hospital Würzburg, Germany for a large-scale study [23]. For our study, we firstly included 32 FMS patients (8 per cluster) and blood samples of 25 healthy controls that were available from the large-scale study. During the analysis, only data of 31 patients and 16 controls were valid. We included male and female patients ≥18 years, who were diagnosed with FMS according to the ACR criteria of 1990 and 2010 [1, 24]. Subjects with other possible differential diagnoses for pain (e.g. rheumatologic, orthopedic) or with other and additional pain sources (e.g. pain due to arthritis) were excluded. Further exclusion criteria were diabetes mellitus, polyneuropathy, psychiatric conditions, cancer, epilepsy, drug and alcohol abuse, allergies to local anaesthetics, abnormalities in routine blood tests, and ongoing legal issues (e.g. regarding health assurance). Our study was approved by the Würzburg Medical School Ethics Committee (No. 135/15). All study participants provided written informed consent before enrollment. Data on clinical examination, detailed laboratory and electrophysiological measurements are summarized in S1 Table (see S1 Table).

### Questionnaire assessment for clinical data

All patients and controls underwent neurological examination and were assessed with questionnaires for pain, impairment due to FMS symptoms, and psychopathological variables such as depression, anxiety, and pain catastrophizing. We used the German versions of the following questionnaires: The Neuropathic Pain Symptom Inventory (NPSI-G) [25], the Graded Chronic Pain Scale (GCPS) [26], the Fibromyalgia Impact Questionnaire (FIQ) [27], the Center of Epidemiological Studies General Depression Scale (CES) [28], the State-Trait Anxiety Inventory (STAI-G) [29], the Pain Catastrophizing Scale (PCS) [30], and the Childhood Trauma Questionnaire (CTQ) [31].

### Blood withdrawal, white blood cell extraction, and miRNA isolation

Venous whole blood was drawn from all patients and 25 healthy controls as previously described in [12]. Data of 31 patients and 16 controls were valid for the analysis. After the extraction of the white blood cell (WBC) fraction, all samples were stored at -80˚C until further processing. The manufacturer’s protocol of the miRNeasy Mini kit (Qiagen, Hilden; ermany) was followed to isolate miRNA from WBC samples and the RNA concentration was measured by Nanodrop Photometer Pearl^®^ (Implen, München, Germany).

### Selection criteria for candidate miRNA

MiRNA were selected based on literature search. We used “inflammation”, “chronic stress”, and “resilience” as search terms and cross-compared results for miR103a-3p, miR107, miR130a-3p, and miR125a-5p in public data bases (miRbase [32], NCBI [33]). We then compared our results with those of a previous miRCURY LNA miRNA array taken by the profiling service of Exiqon (Exiqon Services, Vedbaek, Denmark) [9] and decided on four miRNA that had survived Benjamini-Hochberg correction (p < 0.05) and showed ≥ 60fold log-fold change.

### cDNA synthesis and SYBR green real-time PCR (qRT–PCR)

Five ng of the isolated miRNA was appropriate for the cDNA synthesis which was conducted by following the manufacturer´s protocol of the miRCURY LNA RT Kit (Qiagen, Hilden, Germany). Before starting the SYBR Green qRT-PCR, four mL of synthesized cDNA was diluted (1:80). The miCURY LNA miRNA PCR Assay (Qiagen, Hilden, Germany) provided specific reference primer sets for amplification of the selected miRNA. Specific miCURY LNA assays were labeled with the following assay IDs (in brackets) for all miRNA assessed: hsa-miR 107 (5’AGCAGCAUUGUACAGGGCUAUCA, MIMAT0000104), hsa-miR 103a-3p (5’AGCAGCAUUGUACAGGGCUAUGA, MIMAT0000101), hsa-miR 130a-3p (5’CAGUGCAAUGUUAAAAGGGCAU, MIMAT0000425), hsa-miR 125a-5p (5’UCCCUGAGACCCUUUAACCUGUGA, MIMAT0000443). Five seconds rRNA (5S) was used to normalize the expression level of the derived miRNA and was received as PCR assay (YP00203955). Each target sample was quantified in triplicate, the 5sRNA was measured in duplicate. RNA free water was used as negative control. A previous run of the control samples determined a calibrator as reference sample to ensure the inter-plate comparability. The relative gene expression was evaluated by the delta-delta CT method [34]. This method directly uses the CT value (threshold cycle at which the fluorescence level reaches a certain amount) to calculate fold changes in miRNA gene expression among groups related to its reference sample and normalized by its individual 5sRNA expression. Lower deltaCT values represent sample detection at earlier PCR cycles and indicate higher gene expression. We calculated 1/deltaCT to illustrate higher values as higher gene expression (compare [35]).

### Statistical analysis

IBM SPSS Statistics 26 software (Ehningen, Germany) was used for statistical analysis and GraphPad Prism (San Diego, CA, USA) for the graphical design. Data distribution was tested with the Shapiro-Wilk test and by observing data histograms. Non-normally distributed data of the questionnaires NPSI, GCPS, CTQ (subscales “sexual abuse”, “physical abuse”, and “trivialization”), and of the gene expression analysis are given as median (MED) and range (R). The normally distributed data of all other questionnaires are presented as mean (M) and standard deviation (SD). Spearman correlation tests analyzed correlation between relative gene expression and selected clinical scores of questionnaires. One-Way ANOVA and post-hoc tests (Games-Howell) analyzed group differences between patients and controls, and among cluster. Data are significant at p < 0.05.

## Results

Complete data sets of 31 patients and 16 controls were suitable for PCR analysis and questionnaire assessment (see S1 Fig).

### Group characteristics

Table 1 summarizes demographic characteristics and questionnaire data of the study cohort.

**Table 1:** Demographic characteristics and questionnaire data.

	Maladaptive cluster	Adaptive cluster	Vulnerable cluster	Resilient cluster
**N**	8	8	7	8
**Age**	52.4	53.7	47.2	49.4
NPSI-D*	47 ± 0.52	28 ± 0.21	42 ± 0.34	48 ± 0.18
**FIQ-D**	50.1 ± 9.8	35.8 ± 3.4	54.9 ± 9.4	48.9 ± 5.6
GCPS_grade*	2 ± 2	2 ± 2	2 ± 2	2 ± 0
**CES-D**	21.1 ± 8.9	13.0 ± 7.4	30.2 ± 8.3	17.1 ± 3.5
**PCS-D**	21.9 ± 6.8	8.5 ± 6.8	29.6 ± 6.0	10.2 ± 3.2
**STAI-S**	47.0 ± 8.6	39.5 ± 7.3	63.6 ± 7.3	38.7 ± 9.7
**STAI-T**	47.1 ± 8.2	43.3 ± 7.5	62.2 ± 8.3	37.8 ± 7.7
**CTQ-D**				
**[N = 22]**^**a**^	**[7]**	**[4]**	**[5]**	**[6]**
**emotional neglect**	10.3 ± 5.4	7.3 ± 1.3	12.4 ± 6.0	8.33 ± 2.7
sexual abuse*	7 ± 17	5.5 ± 4	7 ± 11	5.7 ± 8
physical abuse*	9.7 ± 7.0	5.0 ± 0.0	6.2 ± 2.7	5.7 ± 1.0
**emotional abuse**	13.6 ± 3.3	10.5 ± 4.2	16.0 ± 4.1	14.2 ± 3.2
**physical neglect**	7.4 ± 2.1	6.5 ± 2.4	8.8 ± 4.3	8.5 ± 1.8
trivialization*	0 ± 1	0 ± 0	0 ± 0	0 ± 0

*not normally distributed data, MED (= median) ± R (= range); ^a^ CTQ-D questionnaire was added during the study and represents data of 22 patients.

Based on data obtained in our previous study [12], a cluster classification is included in Table 1 reflecting cluster-specific differences.

### Relative gene expression of miR103a-3p, miR107, miR125a-5p, and miR130a-3p in FMS patients and healthy controls

**Fig 1:**
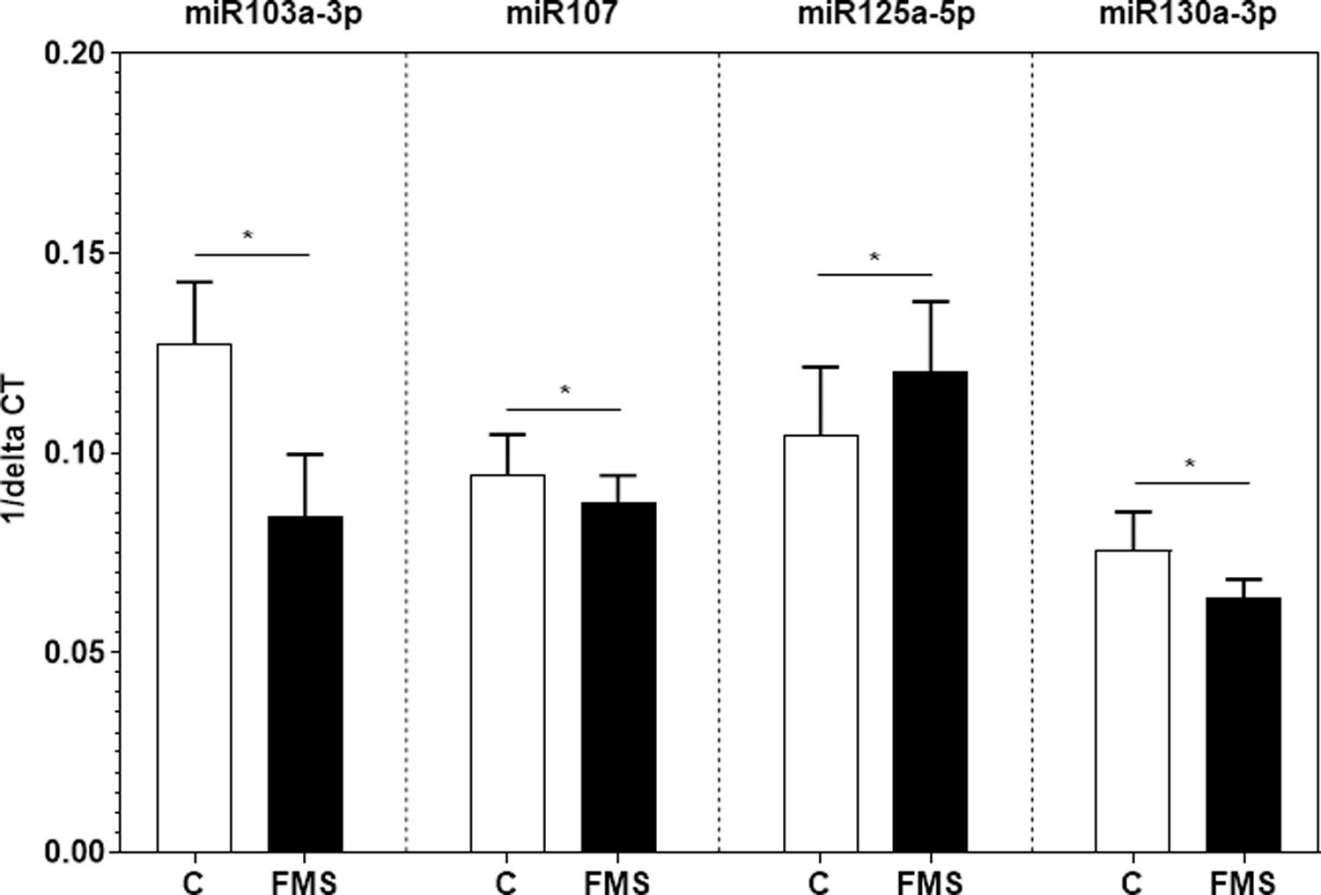
Relative gene expression of four selected miRNA in WBC samples of FMS patients and healthy controls. Boxplots show ΔCT values of miR103a-3p, miR107, miR125a-5p, and miR130a-3p of patients with FMS and healthy controls normalized to the housekeeping gene 5sRNA. Data are presented as 1/ΔCT. Intergroup differences were seen for miR103a-3p, miR107, miR125a-5p and miR130a-3p (p < 0.05). Abbreviations: C = controls; CT = cycle threshold; FMS = patients with fibromyalgia syndrome.

One-way ANOVA and post-hoc tests revealed that miR103a-3p, miR107, miR125a-5p and miR103a-3p were differently expressed when comparing FMS patients and healthy controls (p < 0.05, Fig 1).

Relative gene expression of miR103a-3p, miR107, and miR130a-3p was lower in patients with a large, small and medium effect size, while the relative gene expression of miR125a-5p was higher in FMS patients with a medium effect (Table 2 and Fig 1).

**Table 2:** Effect sizes underline the large differences in miR103a-3p between unfavorable and favorable cluster.

	d	r	Comment**
Cluster*			
**miR103a-3p**			
BC*	3.6	0.9	large
AC*	2.9	0.8	large
DA*	-3.0	-0.8	large
BD*	3.6	0.9	large
DC*	-0.3	-0.2	small
**miR107**			
A*control	-1.2	0.5	medium
**FMS vs. control**			
miR103a-3p	-2.8	-0.8	large
miR107	-0.8	-0.4	small
miR125a-5p	1.0	0.5	medium
miR130a-3p	-1.6	-0.6	medium

*Capitals symbolizing cluster A (maladaptive), B (adaptive), C (vulnerable), and cluster D (resilient) and the calculated effect size r and Cohen’s d between them

**Evaluation of effect size regarding following grades: 0.2 = small, 0.5 = medium, 0.8 = large

Calculated with effect size calculator of the University of Colorado, https://lbecker.uccs.edu/

The effect sizes show a large effect for the difference in gene expression of miR103a-3p among all cluster, except for the resilient and the vulnerable cluster. Even the effect between the entire cohort and the control group was calculated as large. The slight decrease in miR107 of the maladaptive cluster differed to the control group with a medium effect but was small between the patient and control group. It is of note that the variance of relative gene expression for all measured miRNA was high in the patient cohort.

### Relative gene expression of miR103a-3p, miR107 and miR130a-3p in FMS cluster

**Fig 2:**
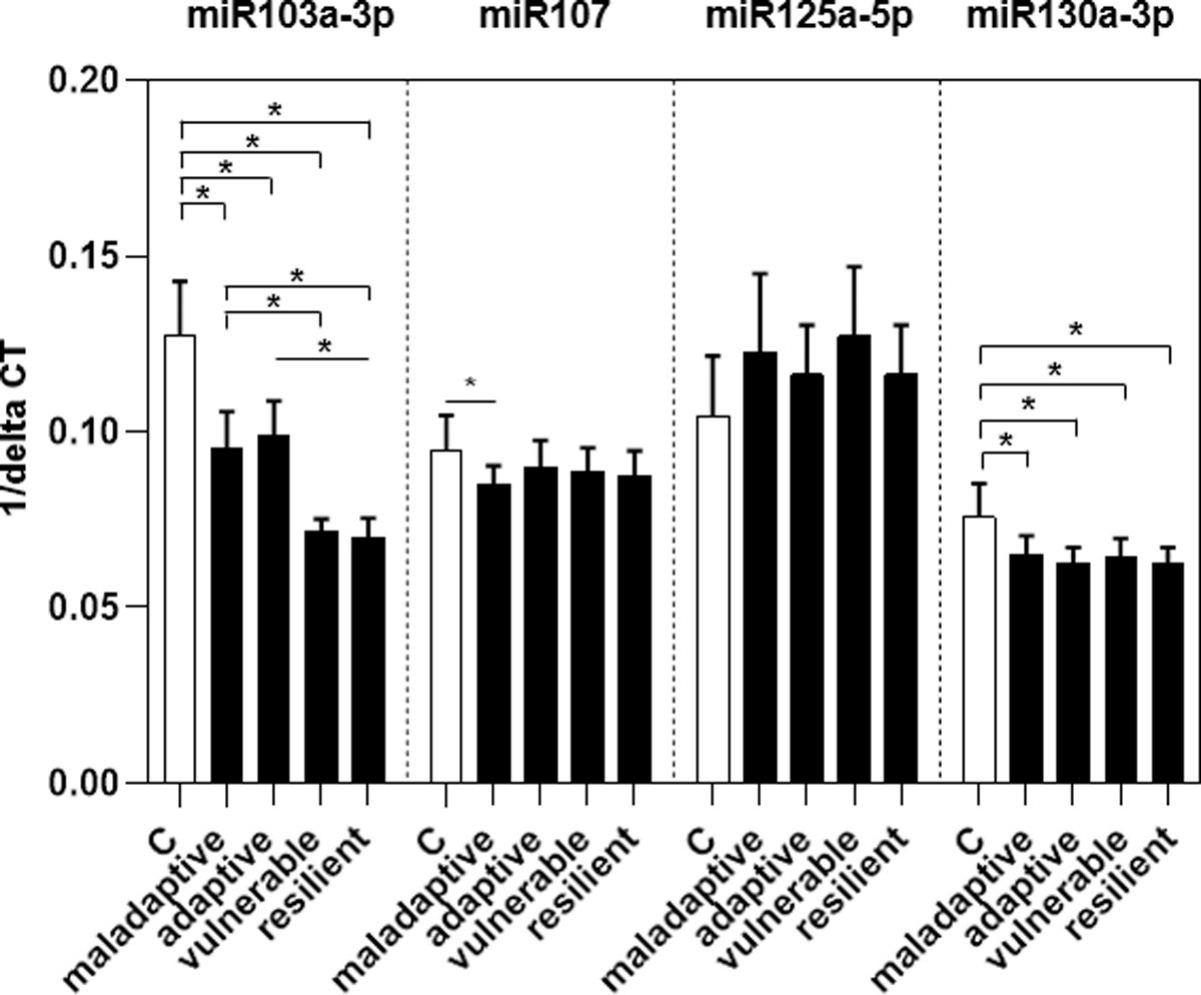
Relative gene expression of four selected miRNA in WBC samples of FMS patients illustrated regarding their cluster division in maladaptive, adaptive, vulnerable, and resilient cluster. Data are presented as 1/ΔCT. Differences among cluster are detected for miR103a-3p (p < 0.05). Abbreviations: C = controls.

Post-hoc tests revealed that only miR103a-3p was differently expressed among FMS patient clusters (Fig 2).

For miR107 and miR130a-3p only differences were apparent between the control group and individual clusters. Relative gene expression of miR125a-5p was different between patients and controls with a medium effect size (Table 2), while no intergroup differences were detected among clusters. Patients assigned to the adaptive cluster had higher miR103a-3p gene expression (MED = 0.1, R = 0.03) than patients in the vulnerable cluster (MED = 0.07, R = 0.01, p < 0.05) and the resilient cluster (MED = 0.07, R = 0.02, p < 0.05). Patients in the maladaptive cluster had a higher expression of miR103a-3p (MED = 0.09, R = 0.03) compared to those in the vulnerable cluster (MED = 0.07, R = 0.01) and the resilient cluster (MED = 0.07, R = 0.02, p < 0.05). In contrast, miR107 expression was lower in patients within the maladaptive cluster (MED = 0.09, R = 0.01) compared to the control group (MED = 0.1, R = 0.03, p < 0.05) with a medium effect size of 0.5 (Table 2). The differences in relative gene expression of miR130a-3p between the control group and each cluster (p < 0.05) were of a medium effect size (Table 2).

### Association between miRNA expression and clinical scores

Spearman correlation analysis indicated an association between the expression of miR103a-3p with the FIQ sum score (r = -0.4, p < 0.05), and the expression of miR107 and the subscale “physical abuse” of the CTQ-D questionnaire (r = -0.5, p < 0.05; Table 3).

**Table 3:** Correlation between relative gene expression of miRNA and clinical scores within the patient cohort (N = 22).

miR	Clinical score	r*
miR103a-3p	FIQ	-0.4**
miR107	CTQ physical abuse	-0.5**
miR125a-5p	PCS	0.4**

*Spearman coefficient r

**Significance level p < 0.05

Gene expression of miR125a-5p was associated with the sum score of the PCS questionnaire (r = 0.4, p < 0.05).

## Discussion

We report a subgroup-specific miRNA signature in a cluster of FMS patients that is associated with adaptive coping in terms of active, problem-focused, and cognitive coping resulting in flexibility and adaptability during aversive life periods. After validation and further extension, this signature may serve as an identification code in research of further resilience factors in FMS.

Our data show lower expression levels of miR103a-3p, miR107, and miR130a-3p in patients compared to healthy controls, except for miR125a-5p. Several other studies on miRNA expression in biomaterial obtained from FMS patients revealed lower expression levels in FMS patients compared to healthy controls [7–9, 11, 36]. Here we focused on cluster-specific differences in gene expression, which were found for miR103a-3p. However, since the differences in gene expression for miR130a-3p were seen between the control group and each cluster, but not exclusively among clusters, and no intergroup differences among cluster for miR125a-5p were detected, we focused on miR103a-3p and miR107. Mean miR107 gene expression did not differ among clusters but tended to be higher in the adaptive cluster. Since miR107 forms a family with miR103 with similar physiological functions [37], we included miR107 to the proposed regulatory model.

**Fig 3:**
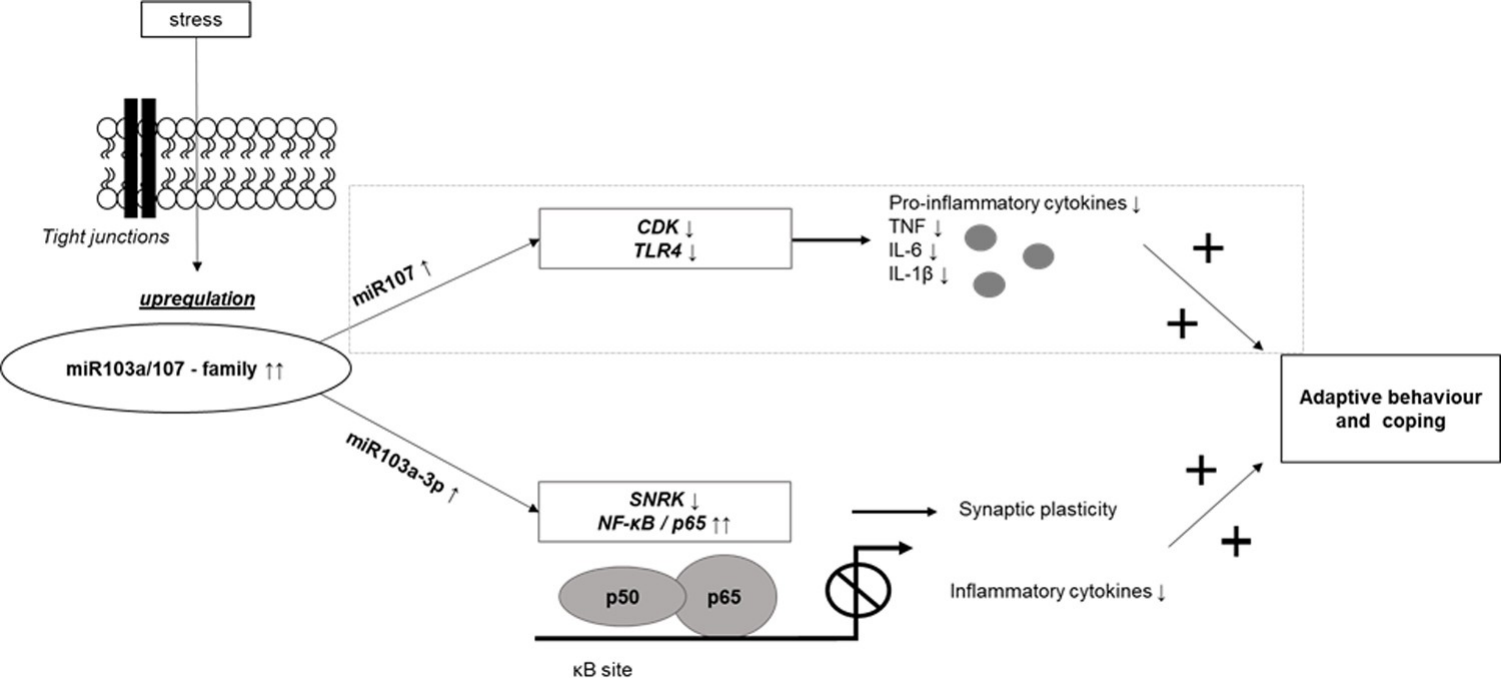
Synopsis of a speculative regulatory process of adaptive behavior in a cluster of FMS patients. Upregulated miRNA expression of miR103a-3p might be responsible for a regulatory cascade of SNRK and NF-κB signalling leading to adaptation on FMS symptoms in a subgroup of FMS patients. Based on the fact of forming a miRNA–family with miR103a-3p and the slight increased gene expression in our adaptive FMS cluster, the speculative potential signalling cascade of miR107 is included and marked as only based on literature data and a trend in our data. Abbreviations: ↑ symbolizes upregulation, ↓ symbolizes downregulation; + symbolizes the positive and resilience / adaptation promoting effect; CDK = cyclin dependent kinases; IL6 = interleukin 6; IL-1β = interleukin 1beta; NF-κB = nuclear-factor kappa B subunit protein 65; SNRK = sucrose non-fermentable serine/threonine-protein kinase; TLR4 = toll-like receptor 4; TNF = tumor necrosis factor-alpha.”

In our previous study [12], the adaptive profile was characterized by active problem- and emotion-focused coping resulting in low scores in disability, depression, anxiety, pain catastrophizing, and early life stress (Table 1). The current study shows an association between the adaptive character and a higher expression of miR103a-3p in this cluster. In a speculative model, we outline the relationship between adaptive behavior and the increased expression of miR103a-3p by targeting specific genes that are involved in resilience promoting and inflammatory pathways supported by literature, correlation, and expression data (please see Fig 3).

Early life stress and inflammation may have a huge impact on the development of chronic pain and is reported in several studies among FMS patients [38, 39]. MiR107 is associated with childhood traumatization [15] and plays a regulatory role in inflammation [16, 17] by targeting CDK and TLR4 [18, 19]. Pro-inflammatory cytokines i.e. TNF, interleukin 6 (IL-6), or IL-1β are released by this process [20]. We speculate that higher miR107 gene expression may lead to lower expression of CDK and TLR4 resulting in low pro-inflammatory cytokine levels. A pro-inflammatory profile may lead to depressive behavior [40] whereas low levels of pro-inflammatory cytokines favor permissive behavior [41, 42].

Despite the lacking increase of miR107 gene expression in our patient cohort, we decided to include miR107 into the synopsis. Both form a miRNA family and there are valid data on the influence of miR107 via TLR4 and CDK signalling on adaptation and inflammation. We can only speculate which potential reasons it might have that although miR107 and miR103a-3p belong to the same family, miR107 seems not to have the same influence in our cohort as miR103a-3p. As illustrated in Fig 3, miR107 is influenced by TLR4 and CDK signalling. It might be that both genetic targets are differently expressed in our patient cohort resulting in a less prominent role of miR107 here. As one of our study limitations, we stated that we only did verify the genetic targets by an online tool.

MiR103a-3p is linked to stress and inflammation by regulating the SNRK / NF-κB / p65 signaling pathway [43]. Higher gene expression of miR103a-3p may lead to suppression of sucrose non-fermentable serine/threonine protein kinase (SNRK) and ultimately lead to an over-activation of the transactivating subunit protein 65 (p65) of nuclear factor kappa B (NF-κB). NF- κB was reported to be linked to epigenetic resilience promoting mechanisms [44] and synaptic plasticity resulting in adaptive processes [45].

We report on higher relative gene expression of the miR103a/107 family in an adaptive cluster of FMS patients compared to the vulnerable / maladaptive cluster. This regulatory process may be responsible for adaptation via SNRK / NF-kB and via CDK/TLR4 signalling (Fig 3).

Our study has some limitations: the study cohort is small which was due to the availability of patient biomaterial. The selection of miRNA candidates was based on previously published research [9] and also the endogenous control for the PCR was adopted and not further verified. We did not assess the four proposed genetic targets SNRK, CDK, TLR4, and NF-κB, but stayed with *in silico* verification as genetic targets of the miR103a/107 family by the online tool *TargetScanHuman* [46, 47]. Our findings including the expression level of the suggested genetic targets of the miR103/107-family need to be confirmed in an independent FMS patient group.

## Conclusion

We show an association between higher gene expression of miR103a-3p and miR107 and adaptive coping in a cluster of FMS patients. This miRNA signature might function as a diagnostic profile of FMS subgroups and enable further research on somatic parameters of adaptation and resilience in FMS.

## Supporting information

S1 Fig Flow chart of patient recruitment. (TIF)

S1 Table Data of clinical examination, laboratory, and electrophysiological measurements. BMI = body mass index; NCV = nerve conduction velocity.
(DOCX)
