# Supplementary figures and images for: MiR103a-3p and miR107 are related to adaptive coping in a cluster of fibromyalgia patients
Source: PLoS One. 2020 Sep 17;15(9):e0239286. doi: 10.1371/journal.pone.0239286 (PMC7498021; doi:10.1371/journal.pone.0239286)

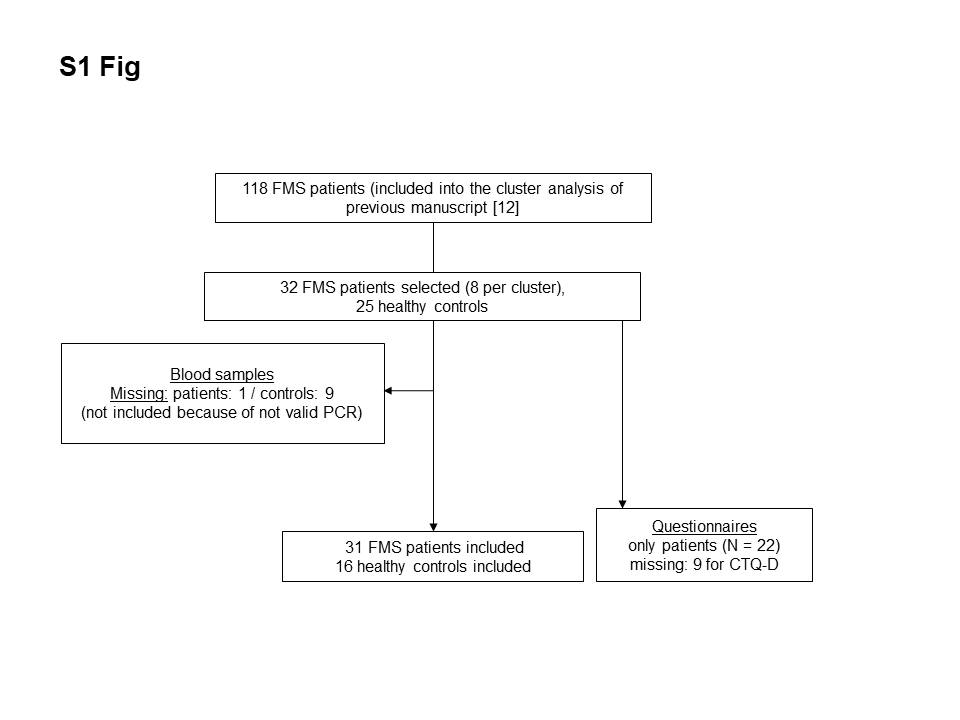

Supplement: S1 Fig — (TIF) [file pone.0239286.s001.tif]
