# Supplementary material for: MiR103a-3p and miR107 are related to adaptive coping in a cluster of fibromyalgia patients
Source: PLoS One. 2020 Sep 17;15(9):e0239286. doi: 10.1371/journal.pone.0239286 (PMC7498021; doi:10.1371/journal.pone.0239286)
Supplement: S1 Table — BMI = body mass index; NCV = nerve conduction velocity. (DOCX) [file pone.0239286.s002.docx]

**S1 Table:** Data of clinical examination, laboratory, and electrophysiological measurements. BMI = body mass index; NCV = nerve conduction velocity.

|  | **Maladaptive**  **[8]** | **Adaptive**  **[8]** | **Vulnerable**  **[8]** | **Resilient**  **[8]** |
| --- | --- | --- | --- | --- |
| **Clinical examination** |  |  |  |  |
| **Gender** |  |  |  |  |
| Female | 8 | 8 | 8 | 8 |
| **Weight [kg]** | 67.6 | 66.69 | 74.5 | 73.5 |
| **Height [cm]** | 159.6 | 163.3 | 167.8 | 164.9 |
| **BMI** | 22.9 | 22.5 | 25.2 | 24.8 |
| **Employment status** |  |  |  |  |
| Regularly working | 3 | 5 | 5 | 3 |
| Sick leave because of pain | - | - | 1 | 3 |
| Retired because of pain | 2 | - | 1 | 2 |
| **Time since diagnosis [years]** | 4.2 | 8.5 | 2.8 | 6.8 |
| **Duration of pain due to the disease [years]** | 12.8 | 16.4 | 12.5 | 11.5 |
| **Pain distribution type** |  |  |  |  |
| Proximal | 3 | 1 | 4 | 1 |
| Distal | - | - | - | - |
| Whole body | 4 | 7 | 3 | 6 |
| **Current pain intensity [scale 0 - 10]** | 6.8 | 4.5 | 6 | 6.3 |
| **Pain character [%]** |  |  |  |  |
| Tearing | 6 | 25 | 9 | - |
| Pressing | 25 | 25 | 50 | 58 |
| Burning | 25 | 25 | 25 | 8 |
| Muscle sourness | 25 | - | 8 | 17 |
| Stabbing | 19 | 25 | 8 | 17 |
| **Psychological/psychiatric treatment** |  |  |  |  |
| Never | 3 | 3 | 3 | 4 |
| Currently | 2 | 2 | 5 | - |
| In the past | 3 | 3 | - | 4 |
| **Electrophysiological measurements** |  |  |  |  |
| **Sural nerve** |  |  |  |  |
| Peak to peak amplitude [µV] | 24.5 | 23.3 | 21.5 | 25.4 |
| NCV [m/s] | 49.3 | 45.7 | 50.0 | 48.6 |
| **Tibial nerve** |  |  |  |  |
| Proximal amplitude [mV] | 14.7 | 15.1 | 16.4 | 16.5 |
| distal amplitude [mV] | 19.1 | 20.3 | 22.0 | 21.5 |
| dmL [ms] | 3.8 | 3.3 | 3.6 | 3.4 |
| NCV [m/s] | 47.6 | 47.1 | 45.8 | 46.5 |
